# On the Use of the Ecraseur for the Removal of Nævoid Growths

**Published:** 1871

**Authors:** James F. West

**Affiliations:** Senior Surgeon to the Queen’s Hospital, and Professor of Anatomy in Queen’s College, Birmingham


Article 148.—*On the Use of the Ecraseur for the Removal of Nævoid Growths.* By James F. West, F.R.C.S., Senior Surgeon to the Queen's Hospital, and Professor of Anatomy in Queen's College, Birmingham.

---

[The Lancet, March 4th.]

Mr. West states the advantages of the ecraseur for the removal of vascular and pendulous tumors have been known to him from practical experience for some years, having frequently by means of it taken away hæmorrhoids, uterine polypi, &c., with perfect success; but he had not employed it for the treatment of nævi, having generally been able to cure them by means of the ligature, the chloride-of-zinc paste, or the injection of the perchloride of iron, until he met with a case in which, the ligature failing entirely, he was induced to try the ecraseur, and as this answered perfectly, he has again employed it on many occasions; but he is far from saying that it should replace all the other methods which have hitherto been used. Each case must be treated on its own merits. Thus there are certain cases in which the setting up of adhesive inflammation, and the consequent obliteration of the vessels supplyiug them by the injection of the perchloride of iron, by vaccination, or the introduction of heated wires into them, may be advantageously employed. But this principle cannot be carried out in many nævi of the face, as a large, dense cicatrix is thereby produced, which is often very unsightly. The simple application of collodion, or of pressure by elastic pads, may cure in slight cases.

The destruction of the nævi by caustics, again, is attended by uncertain results, and the consequent cicatrices are often deep and ugly, from the impossibility of our gauging the distance to which the caustics—as chloride of zinc, nitric acid, &c.—ought to penetrate the tissues.

The ablation of erectile tumors is probably the most perfectly reliable means of treatment, and this may be accomplished either bp enucleation, the ligature, the knife, or the ecraseur.

Piecemeal excision or enucleation is often attended with great loss of blood, even where the adjacent arterial trunks have been compressed as completely as possible; and the little patients who are the ordinary subjects of nævi bear hæmorrhage badly, hence the older surgeons were just in laying it down as a rule that, in removing nævi, it was always proper to cut wide of the tumor, and on no account to cut into its mass.

The introduction of either harelip pins or of ligatures frequently fails to cure; the latter are especially unreliable with venus nævi of large size, owing to their becoming loose, even though the skin around the growths may not have been included in them. The parts daily diminish in size, so that ligatures have to be again and again applied to insure the entire destruction of the tumor. Moreover, ligatures often set up troublesome ulcerations at the base of the nævi, from which occasionally severe hæmorrhage takes place.

The avantages which, in Mr. West's experience, the ecraseur offers are, that hemorrhage is avoided—an important element in all operations, but particularly so with children, and that a linear cicatrix is formed, and a comparatively small wound; and hence that the deformity which, by other operative procedures, will almost of necessity be produced, is prevented or diminished. Chassaignac, also, claims for it that less inflammatory action and less suppuration attend its use than that of the knife; and, consequently that the wounds resulting therefrom heal more readily, and are less likely to be followed by pyæmia. On these latter points Mr. West offers no opinion.

